# Is the duration of dual antiplatelet therapy (DAPT) excessive in post-angioplasty in chronic coronary syndrome? Data from the France-PCI registry (2014–2019)

**DOI:** 10.3389/fcvm.2023.1106503

**Published:** 2023-03-24

**Authors:** A. Mezier, P. Motreff, J. M. Clerc, O. Bar, R. Deballon, T. Demicheli, T. Dechery, G. Souteyrand, A. Py, N. Lhoest, T. Lhermusier, B. Honton, A. Gommeaux, J. Jeanneteau, P. Deharo, H. Benamer, G. Cayla, R. Koning, B. Pereira, J. P. Collet, G. Rangé

**Affiliations:** ^1^ Cardiology Department, Centre Hospitalier Universitaire de Clermont-Ferrand, Clermont-Ferrand, France; ^2^ Cardiology Department, Centre Hospitalier Universitaire de Tours, Tours, France; ^3^ Cardiology Department, Nouvelle Clinique Tourangelle, Saint-Cyr-sur-Loire, France; ^4^ Cardiology Department, Clinique Oréliance, Orléans, France; ^5^ Cardiology Department, Les Hôpitaux de Chartres, Chartres, France; ^6^ Cardiology Department, Centre Hospitalier Jacques Coeur, Bourges, France; ^7^ Cardiology Department, Clinique de l’Europe, Amiens, France; ^8^ Cardiology Departemnt, Clinique Rhéna, Strasbourg, France; ^9^ Cardiology Department, Centre Hospitalier Universitaire de Toulouse, Toulouse, France; ^10^ Cardiology Department, Clinique Pasteur, Toulouse, France; ^11^ Cardiology Department, Hôpital Privé de Bois-Bernard, Bois-Bernard, France; ^12^ Cardiology Department, Clinique Saint Joseph, Trelaze, France; ^13^ Cardiology Department, Centre Hospitalier Universitaire de la Timone, Marseille, France; ^14^ Cardiology Department, Institut Cardiovasculaire Paris Sud, Massy, France; ^15^ Cardiology Department, Centre Hospitalier Universitaire de Nîmes, Nîmes, France; ^16^ Cardiology Department, Clinique Saint Hilaire, Rouen, France; ^17^ Clinical Research and Innovation Direction, Centre Hospitalier Universitaire de Clermont-Ferrand, Clermont-Ferrand, France; ^18^ Cardiology Institute, Hôpital Pitié-Salpêtrière (Assistance Publique-Hôpitaux de Paris), Paris, France

**Keywords:** coronary angioplasty, chronic coronary syndrome, dual antiplatelet therapy, bleeding risk, ischemic risk

## Abstract

**Background:**

while the duration of dual antiplatelet therapy (DAPT) following coronary angioplasty for chronic coronary syndrome (CCS) recommended by the European Society of Cardiology has decreased over the last decade, little is known about the adherence to those guidelines in clinical practice in France.

**Aim:**

To analyze the real duration of DAPT post coronary angioplasty in CCS, as well as the factors affecting this duration.

**Methods:**

Between 2014 and 2019, 8.836 percutaneous coronary interventions for CCS from the France-PCI registry were evaluated, with 1 year follow up, after exclusion of patients receiving oral anticoagulants, procedures performed within one year of an acute coronary syndrome, and repeat angioplasty.

**Results:**

Post-percutaneous coronary intervention (PCI) DAPT duration was > 12 months for 53.1% of patients treated for CCS; 30.5% had a DAPT between 7 and 12 months, and 16.4% a DAPT ≤ 6 months. Patients with L-DAPT (>12 months) were at higher ischemic risk [25.0% of DAPT score ≥2 vs. 18.8% DAPT score ≥2 in S&I-DAPT group (≤12 months)]. The most commonly used P2Y12 inhibitor was clopidogrel (82.2%). The prescription of ticagrelor increased over the period.

**Conclusions:**

post-PCI DAPT duration in CCS was higher than international recommendations in the France PCI registry between 2014 and 2019. More than half of the angioplasty performed for CCS are followed by a DAPT > 12 months. Ischemic risk assessment influences the duration of DAPT. This risk is probably overestimated nowadays, leading to a prolongation of DAPT beyond the recommended durations, thus increasing the bleeding risk.

## Introduction

Coronary angioplasty is a routine treatment for chronic coronary syndrome (CCS) with ischemic lesion. Dual antiplatelet therapy (DAPT) is essential after coronary angioplasty to reduce thrombotic complications (mostly stent thrombosis). Advances in devices and techniques have increased the safety of coronary angioplasty by reducing those complications, with the development of the latest generations of drug eluting stent (DES) in particular (1, 2). These technological improvements have allowed a reduction in DAPT duration. In 2017, the DAPT duration recommended after coronary angioplasty for CCS was shortened to 6 months (3), from 6 to 12 months previously (4). The duration of DAPT should be adjusted for each patient, depending on individual bleeding and ischemic risk (3). Shorter DAPT duration is associated with increased risk of stent thrombosis (ST) and myocardial infarction (MI), while longer DAPT duration is at increased risk of bleeding. The recommended P2Y12 inhibitor in the context of CCS for post-coronary angioplasty is clopidogrel, in addition to aspirin (3–5).

We analyzed the real durations and component drugs of DAPT in post coronary angioplasty for CCS between 2014 and 2019, within the France PCI registry.

## Material and method

### France PCI registry

Data from the France PCI registry were used over a period of 6 years (2014–2019). France PCI is a national registry that aims to collect data on all coronary angiography and coronary angioplasty activities performed in France (6). The registry included 6 angioplasty sites in 2014, and 15 in 2019. 150 variables are systematically collected for each coronary angiography and/or coronary angioplasty procedure. This includes epidemiological, clinical, pre-hospital and procedural data, as well as a follow-up of the hospital stay and a follow-up at 1-year post-procedure. The pre-hospital and procedural data are collected by the operator and automatically extracted from the reports using software (Cardioreport®, Hémolia®), thus allowing an exhaustive collection of data >97% (7). The hospital follow-up data are collected from the patient's medical file. The follow-up at 1 year [including collecting the effective duration and composition of the DAPT, ischemic event or bleeding event (classification BARC)] is done by phone contact with the patient. These data are then entered by dedicated on-site Clinical Research Associates or Clinical Study Technicians. Data of procedures from 2014 to 2019 and their 1-year follow-up were analyzed for this study.

### Inclusion and exclusion criteria

We focused on coronary angioplasty procedures performed in the context of CCS amongst all of the procedures included in the France PCI registry. Procedures performed in the following contexts were considered as coronary angioplasties for CCS: planned angioplasty, stable angina, asymptomatic patient with positive ischemia test, preoperative cardiac and noncardiac surgery, pre Transcatheter Aortic Valve Implantation (TAVI), evaluation coronary angiography (left ventricular dysfunction, flow fractional reserve, optical coherence tomography etc.), and heart failure. Procedures performed in patients receiving oral anticoagulation before coronary angioplasty or within the year after PCI were excluded. Indeed, the co-administration of antiplatelet therapy and oral anticoagulant is subject to specific strategies (3, 8).

Procedures performed on patients with acute coronary syndrome (ACS) within the year before or after coronary angioplasty for CCS were deliberately excluded. The occurrence of ACS impacts the nature and duration of DAPT, leading to a bias at the time of data collection. In the event of iterative angioplasty for CCS over a 1-year period, only the last procedure was retained. Procedures in which the duration of DAPT was unknown were also excluded, as were patients who were not followed-up on or were deceased.

DAPT durations were considered short (S-DAPT) when less than or equal to 6 months, intermediate (I-DAPT) between 7 and 12 months inclusive, and long (L-DAPT) if they exceeded 12 months. The short and intermediate durations were pooled (S&I-DAPT) in order to be compared with the long durations.

The DAPT score was calculated from the France-PCI registry data according to the following criteria: 1 point each for MI at presentation (value = 0 for entire cohort because of inclusion criteria), prior MI or PCI, diabetes, stent diameter less than 3 mm, smoking, and paclitaxel-eluting stent (value = 0 for entire cohort because no Paclitaxel stents were implanted); 2 points each for low ejection fraction (<30%) and vein graft intervention; −1 point for age 65–74 years; and −2 points for age 75 years or older (9).

### Statistical analyses

Patient characteristics were described by numbers and percentages for categorical variables and by the mean and standard deviation or median and interquartile range, with respect to their statistical distribution, for variables of a quantitative nature. Comparisons between groups (≤ and >12 months) concerning quantitative variables considered the Student t test or the Mann–Whitney test when the conditions for application of the t test were not met. The hypothesis of normality was studied by the Shapiro–Wilk test, and that of equality of variances by the Fisher–Snedecor test. Chi-square and Fisher exact tests were used for comparisons of categorical variables between groups. As the unit of analysis was the angioplasty procedure (several measures for a same patient), random-effects models were performed as sensitivity analyses in order to take into account between and within patient variability (as random-effect). The results and the findings were not impacted. A sensitivity analysis was performed in order to evaluate the representativeness of our sample comparing it with patients excluded from this analysis due to missing data or lack of information concerning lost to follow-up (Supplementary Material). All statistical analyses were performed with Stata software (version 15, StataCorp, College Station, United States) for a two-sided type I error at 5%.

## Results

**Figure 1:**
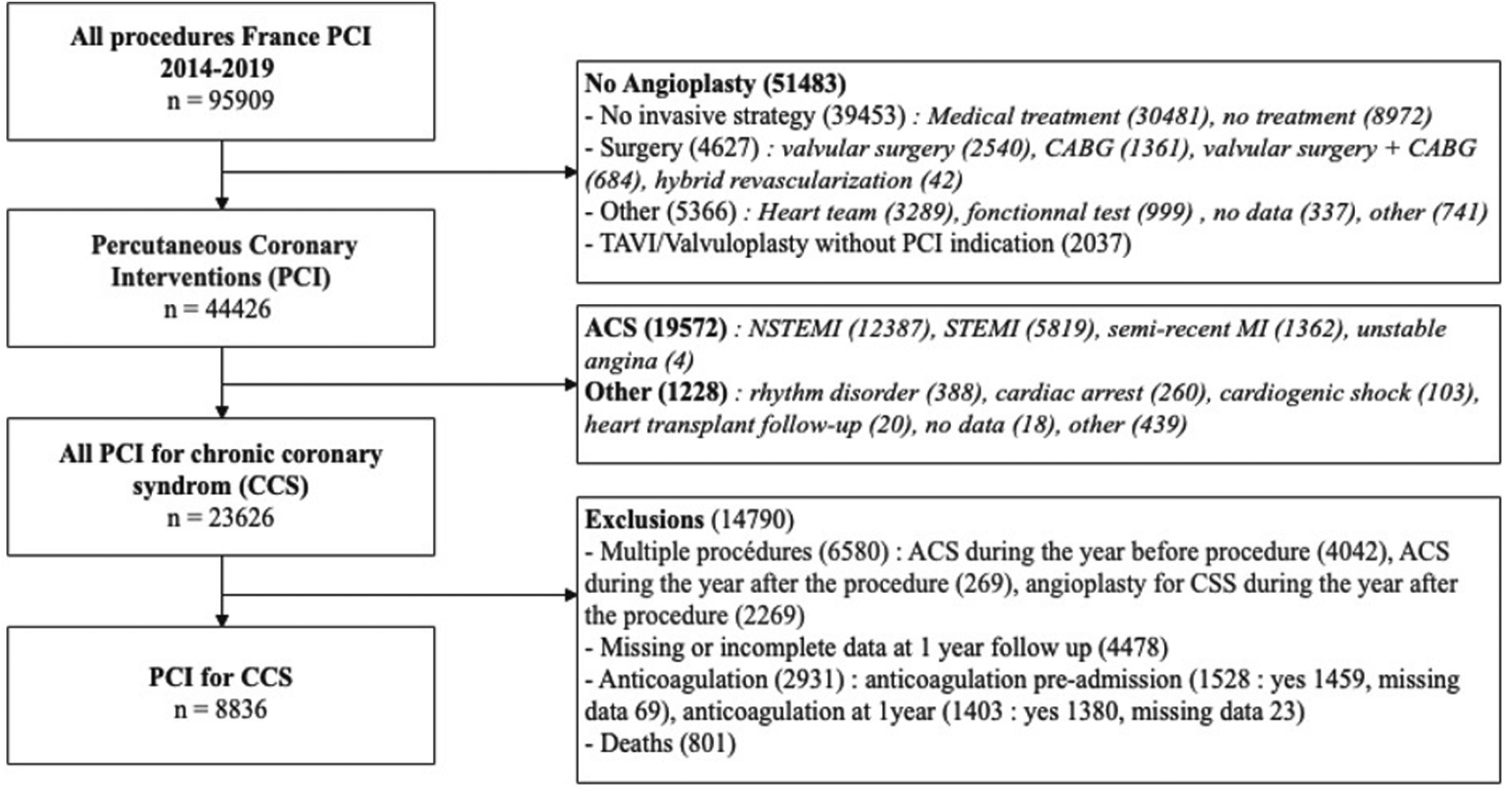
Flow chart. ACS, acute coronary syndrome; CABG, coronary artery bypass graft; CCS, chronic coronary syndrome; MI, myocardial infarction, NSTEMI, non ST-segment elevation myocardial infarction; PCI, percutaneous coronary intervention; STEMI, ST-segment elevation myocardial infarction; TAVI, Transcatheter aortic valve implantation.

**Figure 2:**
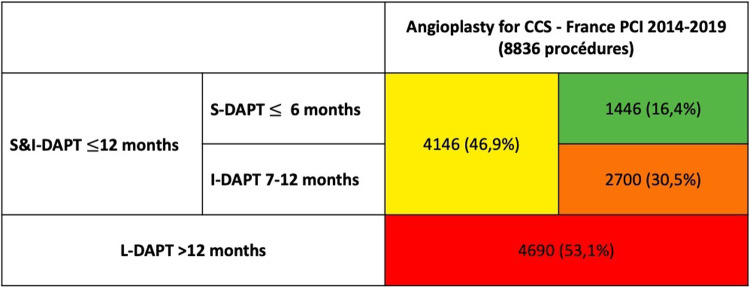
DAPT duration after coronary angioplasty for CCS in the France PCI registry (2014–2019) [*n* = number of procedures (%)]. CCS, chronic coronary syndrome; S-DAPT, short dual antiplatelet therapy; I-DAPT, short & intermediate dual antiplatelet therapy; L-DAPT, long dual antiplatelet therapy.

**Figure 3:**
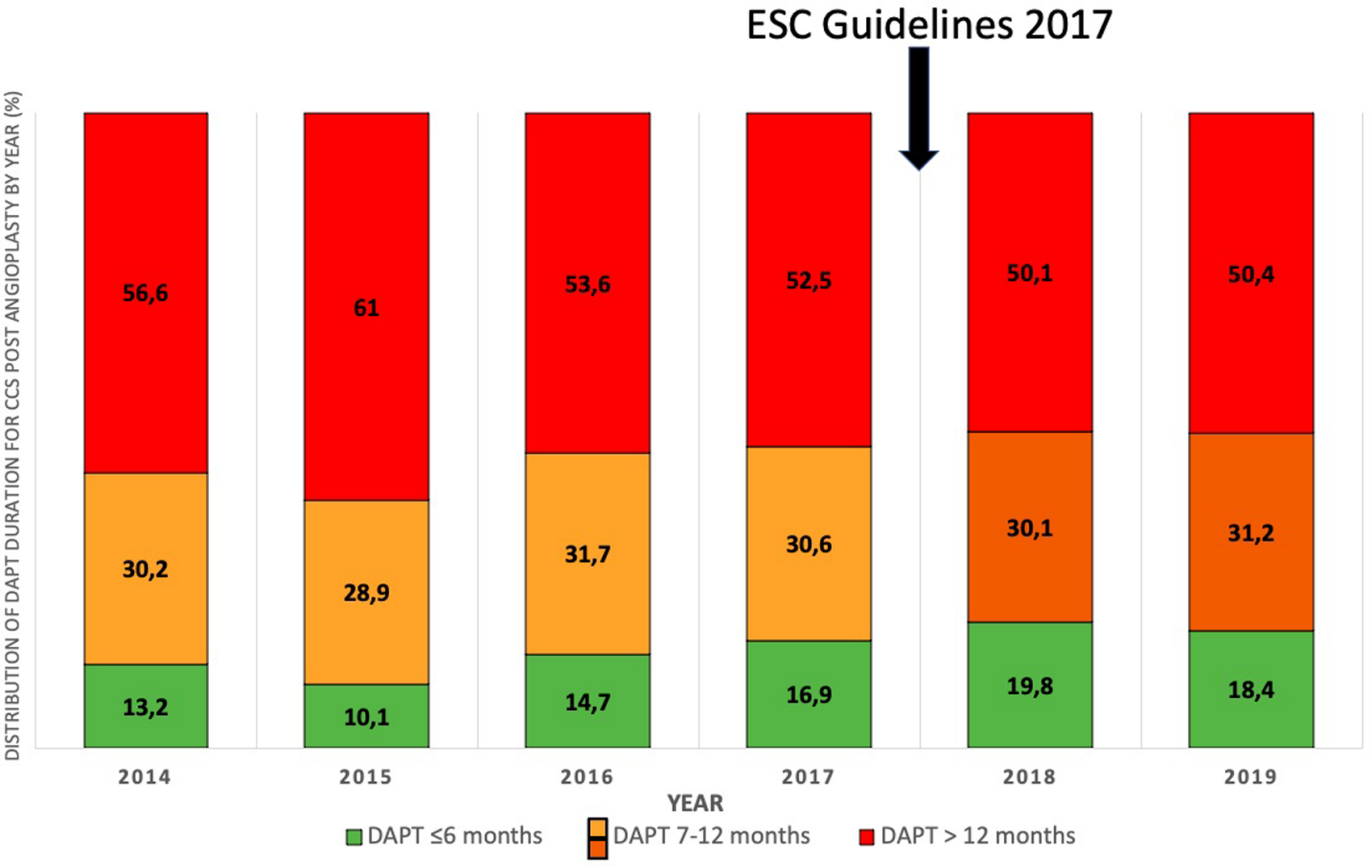
Distribution of DAPT duration for CCS post angioplasty by year (%) (2014–2019). DAPT, dual antiplatelet therapy.

Over the period 2014–2019, 95.909 procedures were collected in France PCI including 44,426 coronary angioplasty. Among them, 23.626 were coronary angioplasty for CCS. After applying exclusion criteria, we studied 8.836 angioplasty procedures for CCS (Figure 1). The characteristics of the population are detailed in Table 1. The mean age was 68.6 years (±10.4) with a predominantly male population (77.6%). The duration of DAPT of those 8.836 angioplasty procedures for CCS is shown in Figure 2. 4.146 (46.9%) were followed by S&I-DAPT, of which 1.446 (16.4%) ≤ 6 months and 2,700 (30.5%) between 7 and 12 months. 4.690 procedures (53.1%) were followed by a L-DAPT. The evolution of post-angioplasty DAPT durations is illustrated in Figure 3. DAPT durations are stable even though L-DAPT decreased from 56.6% in 2014 to 50.4% in 2019 it is still dominant compared to S&I-DAPT.

**Table 1:** DAPT durations according to population characteristics.

	S&I-DAPT (≤12 months) *n* = 4146	L-DAPT (>12 months) *n* = 4690	Total *n* = 8836	*p*-value
**Cardiovascular risk factors**				
Age, mean (SD), year	68.9 (±10.4)	68.3 (±10.5)	68.6 (±10.4)	0.012
Male gender (%)	3,149 (75.9)	3,708 (79.1)	6,857 (77.6)	<0.001
Overweight (BMI >25) (%)	2,766 (66.7)	3,293 (70.2)	6,059 (68.6)	0.005
Hypertension (%)	2,558 (61.7)	2,988 (63.7)	5,546 (62.8)	0.043
Dyslipidaemia (%)	2,345 (56.6)	2,822 (61.2)	5,167 (58.5)	<0.001
Smoking (%)	1,925 (46.4)	2,293 (49.0)	4,218 (47.7)	0.018
Active smoker (%)	567 (13.7)	776 (16.5)	1,343 (15.2)	<0.001
Diabetes (%)	1,207 (29.1)	1,605 (34.3)	2,812 (31.9)	<0.001
Non-insulin requiring (%)	997 (24.0)	1,227 (26.2)	2,224 (25.2)	
Insulin-requiring (%)	210 (5.1)	378 (8.1)	588 (6.7)	
Coronary heredity (%)	1,088 (26.2)	1,148 (24.5)	2,236 (25.3)	0.084
**Cardiovascular history**				
Coronary angioplasty (%)	1,461 (35.2)	1,979 (42.2)	3,440 (38.9)	<0.001
Myocardial infarction (>1 year) (%)	570 (13.8)	752 (16.1)	1,322 (15.0)	0.003
Stroke (%)	130 (3.0)	132 (2.8)	262 (3.0)	0.373
Hemorrhage (%)	10 (0.2)	11 (0.2)	21 (0.2)	0.848
Peripheral vascular pathology (%)	538 (13.0)	676 (14.4)	1,214 (13.7)	0.054
Severe renal failure (%)	83 (2.0)	70 (1.5)	153 (1.7)	0.066
Severe left ventricular dysfunction (%)	105 (2.7)	182 (4.1%)	287 (3.4)	<0.001
DAPT score, median (Quartile)	0 (−1; 1)	1 (0; 2)	0 (0; 1)	<0.001
DAPT score ≥2 (%)	780 (18.8)	1,174 (25.0)	1,954 (22.1)	<0.001

*n*, number of patients (%); SD, standard deviation; Severe left ventricular dysfunction = LVEF, left ventricular ejection fraction <30%; Severe renal failure** **= creatinine > 200 µmol/L.

Patients receiving L-DAPT were significantly younger (68.3 vs. 68.9 years, *p* = 0.012) and more often men (79.1% vs. 75.9%, *p* < 0.001) (Table 1). Among the cardiovascular risk factors, diabetics were more represented in the L-DAPT group (34.3% vs. 29.1%, *p* < 0.001). The same goes for dyslipidemia (61.2% in the L-DAPT group vs. 56.6% in the S&I-DAPT group, *p* < 0.001), smoking (49.0% vs. 46.4%, *p* < 0.001), hypertension (63.7% vs. 61.7%, *p* = 0.043) or overweight (BMI ≥ 25 kg/m^2^) (70.2% vs. 66.7%, *p* = 0.005) with a mean BMI in the population of 27.6 (±4.71). There was no significant difference for coronary heredity.

Regarding cardiovascular history, only coronary angioplasty and prior MI (>1 year) were significantly more frequent in the L-DAPT group (42.2% vs. 35.2%, *p* < 0.001; and 16.1% vs. 13.8%, *p* = 0.003, respectively). L-DAPT was significantly more common in cases of severe left ventricular ejection fraction (LVEF < 30%) (4.1% vs. 2.7%, <0.001).

**Figure 4:**
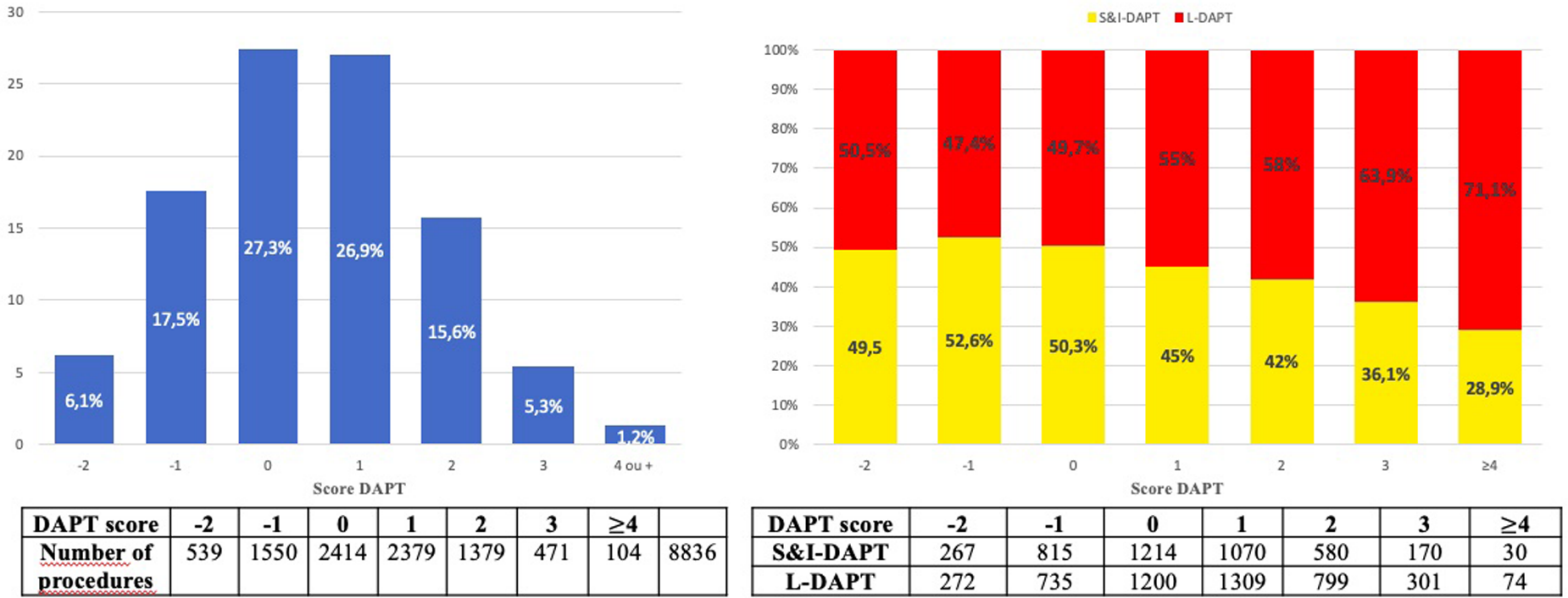
Distribution of the DAPT score (**A**). Distribution of dual antiplatelet therapy duration according to DAPT score (**B**)*.* DAPT, dual antiplatelet therapy; S&I-DAPT, short and intermediate dual antiplatelet therapy (≤12 months); L-DAPT, long dual antiplatelet therapy (>12 months).

The distribution of DAPT score and the distribution of DAPT durations according to this score are shown in Figures 4.

Patients with a high DAPT score ≥ 2 at the time of the procedure (22.1%) significantly more often received prolonged treatment (25.0% L-DAPT vs. 18.8% S&I-DAPT, *p* < 0.001). The proportion of L-DAPT increased with the DAPT score, reaching more than 70% for the highest ischemic risk. Nevertheless, 75% of patients who received L-DAPT did not have a high DAPT score (≥2) at the time of the angioplasty procedure for CCS.

Durations of DAPT according to procedural data and hospital events and 1-year follow-up are detailed in Table 2. Multi-vessel patients are more frequently treated with L-DAPT (62.1% vs. 55.7%, *p* < 0.001), as well as patients with proximal lesions (62.3% vs. 57.1%, *p* < 0.001) or chronic total occlusion (CTO) (24.9% vs. 22.4%, *p* = 0.006). Long length (>20 mm) and small diameter (<3 mm) lesions were preferentially treated with L-DAPT. Regarding procedural data, there were significantly more multiple angioplasties in the L-DAPT group [18.8% number of dilated arteries >1 in the L-DAPT group vs. 15.7% (*p* < 0.001)], 40.6% number of dilated sites >1 in the L-DAPT group vs. 36.8% (*p* < 0.01), 43% number of stents implanted >1 in the L-DAPT group vs. 40% (*p* = 0.02). DAPT times were longer with stents of diameter < 3 mm (L-DAPT 49.9% vs. S&I-DAPT 47.2%, *p* = 0.012). 90.5% of angioplasties were performed with drug eluting stents (DES), with no significant difference (90.5 in S&I-DAPT group vs. 90.4% in L-DAPT group (*p* = 0.32).

**Table 2:** DAPT durations according to procedural data, in-hospital events and 1-year follow-up.

	S&I-DAPT (≤12 months) *n* = 4146	L-DAPT (>12 months) *n* = 4690	Total *n* = 8836	*p*-value
**Lesion characteristics**				
Number of vessel(s) affected (%)				<0.001
Monotruncular	1,815 (43.8)	1,747 (37.2)	3,562 (40.3)	
Pluritruncal	2,309 (55.7)	2,911 (62.1)	5,220 (59.1)	
Isolated left main	22 (0.5)	32 (0.7)	54 (0.6)	
Presence of proximal lesion (%)	2,367 (57.1)	2,922 (62.3)	5,289 (59.9)	<0.001
Presence of CTO (%)	928 (22.4)	1,166 (24.9)	2,094 (23.7)	0.006
Presence of CABG (%)	276 (6.7)	397 (8.5)	673 (7.6)	0.001
Artery diameter at lesion <3 mm (%)	2,590 (62.5)	3,157 (67.3)	5,747 (65.0)	<0.001
Lesion length >20 mm (%)	1,767 (42.6)	2,182 (46.5)	3,949 (44.7)	<0.001
**Characteristics of angioplasty**				
Number of dilated arteries >1 (%)	649 (15.7)	881 (18.8)	1,530 (17.3)	<0.001
Number of dilated sites >1 (%)	1,526 (36.8)	1,902 (40.6)	3,428 (38.8)	<0.001
Number of stents implanted >1 (%)	1,649 (39.8)	1,997 (42.6)	3,646 (41.3)	0.007
Stent diameter <3 mm (%)	1,959 (47.2)	2,341 (49.9)	4,300 (48.7)	0.012
Type of stent(%)				0.32
Drug eluting stent (DES)	3,753 (90.5)	4,240 (90.4)	7,993 (90.5)	
Bare metal stent (BMS)	134 (3.2)	126 (2.6)	260 (2.9)	
Drug-coated balloon (DCB)	89 (2.1)	116 (2.4)	205 (2.3)	
Bioresorbable vascular scaffold (BVS)	19 (0.5)	22 (0.5)	41 (0.4)	
Balloon angioplasty alone.	321 (7.7)	398 (8.4)	719 (8.1)	
**In-hospital events**				
Severe bleeding (BARC ≥ 3) (%)	16 (0.39)	6 (0.13)	22 (0.25)	0.015
Stroke (%)	3 (0.07)	2 (0.04)	5 (0.06)	0.76
Ischemic (%)	2 (0.05)	2 (0.04)	4 (0.05)	
Hemorrhage (%)	1 (0.02)	0	1 (0.01)	
**Events at 1 year**				
Severe bleeding (BARC ≥ 3) (%)	96 (2.3)	23 (0.5)	119 (1.3)	<0.001
Stroke (%)	14 (0.34)	16 (0.34)	30 (0.3)	0.87
Ischemic (%)	11 (0.27)	12 (0.26)	23 (0.26)	
Hemorrhage (%)	2 (0.05)	1 (0.02)	3 (0.03)	
Unspecified (%)	1 (0.02)	3 (0.06)	4 (0.05)	

CTO, chronic total occlusion; CABG, coronary artery bypass graft; BARC, Bleeding Academic Research Consortium.

Shorter DAPT times are observed in cases of severe bleeding (BARC ≥ 3) occurring intra-hospital or in the year after angioplasty for CCS.

Composition of DAPT: among the 8.836 procedures included procedures, 127 (0.14%) were followed by aspirin alone at discharge. For the 8.709 procedures followed by DAPT, aspirin was mostly combined with clopidogrel (82.2%), more rarely with ticagrelor (15%) or prasugrel (2.8%). In the S&I-DAPT group, the anti-platelet therapy at 1 year post PCI was mostly aspirin monotherapy (91%), more rarely clopidogrel (7.4%), exceptionally ticagrelor or prasugrel (0.24%). There were no antiplatelet agents after 52 procedures at 1 year after the procedure (1.2%). The prescription of ticagrelor increased over the period (*p* < 0.001) (Table 3).

**Table 3:** Evolution of P2Y12 inhibitors used in post angioplasty for CCS.

	2014	2015	2016	2017	2018	2019	
Clopidogrel	756 (81.9)	812 (83.1)	1,107 (85.6)	1,384 (79.0)	1,509 (83.5)	1,591 (81.3)	7,159 (82.2)
Ticagrelor/Prasugrel Ticagrelor Prasugrel	167 (18.1) 112 (12.1) 55 (6.0)	165 (16.9) 120 (12.3) 45 (4.6)	187 (14.4) 144 (11.1) 43 (3.3)	367 (21.0) 323 (18.4) 44 (2.5)	299 (16.5) 268 (14.8) 31 (1.7)	365 (18.7) 343 (17.5) 22 (1.2)	1,550 (17.8) 1,310 (15.0) 240 (2.8)
	923	977	1294	1751	1808	1956	8709

There was no significant difference in the proportion of patients still on DAPT at 1 year depending on the P2Y12 inhibitor used at discharge [54.1% with clopidogrel, 52.1% with ticagrelor or prasugrel (*p* = 0.16)].

Patients treated with ticagrelor or prasugrel were younger (mean age 64.6 (±10.2) vs. 69.4 (±10.3), *p* < 0.001), more often male (81.4% vs. 76.6%, *p* < 0.001), and overweight (71.7% vs. 67.9%, *p* = 0.012). They were also more likely to be current smokers or with a history of smoking (52.1% vs. 46.7%, *p* < 0.001). They had more history of MI (20.0% vs. 13.8%, *p* < 0.001) and coronary angioplasty (44.8% vs. 37.6%, *p* < 0.001). Patients receiving clopidogrel were more likely to have severe renal failure (1.9% vs. 1.0%, *p* = 0.018), and significantly more hypertension (63.9% vs. 57.2%, *p* < 0.001). Patients treated with ticagrelor or prasugrel were associated with higher DAPT score [DAPT score ≥ 2 in 31.5% of cases vs. 20.2% of cases for clopidogrel (*p* < 0.001)] (Supplementary Material).

In terms of procedural data, multi-vessels coronary disease and isolated left main coronary artery stenosis were not significantly more often treated with ticagrelor or prasugrel than with clopidogrel (60.1% vs. 58.9% and 0.8% vs. 0.6%, *p* = 0.43). The same applies to the treatment of proximal lesions (61.6% vs. 59.5%, *p* = 0.13), dilated arteries >1 (18.4% vs. 17.3%, *p* = 0.27), or presence of CTO (23.0% vs. 23.1%, *p* = 0.93). From the procedural data, only multiple stenting (number of stents implanted >1) was more often associated with the use of ticagrelor or prasugrel (45.0% vs. 40.9%, *p* < 0.01).

## Discussion

In this homogeneous population managed by angioplasty for CCS between 2014 and 2019, more than half of the procedures were followed by a duration of DAPT > 12 months (53.1%), higher than the duration recommended by European guidelines (3–5). No major changes in the duration of DAPT were found during this period, although the proportion of L-DAPT decreased slightly from 56.6% in 2014 to 50.4% in 2019 while S-DAPT rose from 13.2% in 2014 to 18.4% in 2019.

The recommendations are based on studies confirming the progress observed with the latest generation of DES and the substantial decrease in stent thrombosis rate (2, 5). The recommendations published by the ESC in 2017 which proposed to shorten the duration of DAPT post PCI for CCS to 6 months (3), had no impact on the French practices observed in 2018–2019. Moreover, 17.8% of patients are treated with ticagrelor and prasugrel despite the lack of studies demonstrating the superiority of these molecules in this indication (10). In the ESC guidelines their use may be considered (class IIb) in situations with high risk of stent thrombosis based on procedural data (5). Most notably, there has been an increase in the use of ticagrelor from 12.1% in 2014 to 17.5% in 2019. In the national Swedish Coronary Angiography and Angioplasty Registry (SCAAR), the use of ticagrelor post PCI for CCS is also observed in almost a third of cases (period 2013–2020, Swedeheart Annual Report 2020 – SCAAR).

In the procedural data concerning our cohort, only multiple stenting (number of stents implanted >1) was significantly associated with the use of ticagrelor or prasugrel. Furthermore, the ticagrelor/prasugrel subgroup had more clinical ischemic risk factors (male gender, smoking, previous MI and/or angioplasty). Similarly, they had a significantly more frequent DAPT score >2 (31.5% vs. 20.2%; *p* < 0.001). Thus, in practice, the preference for ticagrelor or prasugrel in post angioplasty for CCS would be guided more by patient profile than by procedural data.

There are very few data on actual durations of post-coronary angioplasty DAPT in the real world. Only a few observational studies have looked at DAPT times and their clinical impact, but these data are prior to 2014, with different techniques and materials, or concern post-MI (11–13). The few observations made with similar but underpowered cohort also show a significant proportion of long DAPT, often beyond the recommended durations (14). These results confirm the poor adherence to clinical practice recommendations regarding the duration and composition of DAPT after coronary angioplasty in CCS. Adherence to the guidelines has been shown to correlate with improved prognosis (5, 15). If we extrapolate the results of studies dedicated to the clinical impact of short DAPT on the occurrence of bleeding events without increasing the ischemic risk (16, 17), we can imagine the medico-economic consequences of better compliance with the recommendations (cost of treatment, hemorrhagic morbidity and mortality, etc.).

Through this study, we can measure the interest of having an exhaustive and reliable registry, which would allow us to analyze medical practices in real life, in a field such as post-angioplasty DAPT which is constantly evolving due to the progress of equipment and new knowledge. The reactivity of the analysis of these data reinforces the monitoring of the evolution of interventional cardiology practices and measuring the impact of corrective measures.

The duration of DAPT should be individually determined according to the ischemic and hemorrhagic risk. There are clinical and procedural elements that may prolong DAPT even in the context of CCS (3, 9). However, in our study we found that the actual duration of post-angioplasty for CCS was significantly longer than the recommended duration for more than half of the procedures, which seems to be excessive in relation to the usual proportion of patients who can be considered at high ischemic risk.

We can imagine that old habits and the fear of ischemic complications lead the operators to extend the duration of DAPT, or even to use more powerful drugs like ticagrelor or prasugrel. The perception of the hemorrhagic risk is possibly underestimated by the interventional cardiologist who is less directly involved in the clinical consequences than in the case of thrombotic complications (intracerebral hemorrhages, digestive hemorrhages). Moreover, we can raised the hypothesis of a delay between the publication of the recommendations and their execution. The late discontinuation of one of the two PAAs can also be explained by insufficient dissemination of information to the various health care actors [non-interventional cardiologist, general practitioner (GP)]. In the absence of all the factors required to estimate the ischemic risk and in the absence of bleeding during the period of recommended DAPT, the referring cardiologist or the GP will tend to maintain DAPT beyond the recommended durations in fear of an ischemic event, and in particular an ST, will occur after the cessation of DAPT. The duration of DAPT may not be sufficiently explicit on the discharge prescription. Moreover, the GP is reluctant to stop such treatment without clear instructions from cardiologist. The decline in medical demography has led to a gap in follow-up consultations, which may also explain a delay in the adjustment of treatments and particularly of DAPT.

The DAPT score was originally designed to stratify the ischemic and hemorrhagic risk of each patient in order to adapt the duration of DAPT in patients who had completed 12 months of DAPT without having a major bleeding or ischemic event and who were not on chronic oral anticoagulation. For this study, we calculated it with pre- and in-hospital data. The relevance of this score has been questioned in the assessment of bleeding risk (18) for which it was not recommended. On the other hand, the DAPT score allows the identification of patients at high ischemic risk (19), as recommended in the 2017 ESC recommendations (3), although the predictive value of this score has not been found in large registries (20, 21). In our study, we found that there are more patients with a high DAPT score (≥2) in the L-DAPT group. The higher the DAPT score, the more patients received L-DAPT (Figure 4B). Although the DAPT score was not reported *a priori* and is not widely used in routine practice, its criteria influenced DAPT durations. Although in our study there was a relationship between prolonged DAPT duration and DAPT score, it should be noted that 75% of patients treated with DAPT >12 months did not have a high DAPT score (22). The median DAPT score is lower in our series than in the study by Yeh et al. where the median was 2 (9), highlighting a moderate ischemic risk, due to exclusion of ACS, supporting the statement of an unjustified extension of DAPT. Systematic calculation and scrupulous respect of the DAPT score could be a tool to limit prolonged DAPT and reserve it only for subjects at higher ischemic risk (22.1% of DAPT score >2 in our series, or a rate 2.4 times lower than that of L-DAPT observed).

This study is observational, based on a registry. Although it allows us to analyze data from a large number of patients and procedures, it represents a sample of 15 French centers. Thus, a “center effect” cannot be ruled out in our analysis, even if we have not observed any major disparity in practices between the different centers (annual reports; https://www.francepci.com). It is not possible through a registry to establish a causal link between post-procedural events and the duration or composition of DAPT. The rare ischemic events were excluded in order not to include ACS. Paradoxically, hemorrhagic events are more frequent in short treatment durations, and the occurrence of these hemorrhagic events probably led to the reduction of antithrombotic treatments considered responsible or at least a co-factor. Indeed DAPT is often stopped for monotherapy when bleeding event occurs with DAPT.

The aim of the study is to document practices in relation to the recommendations and not to discuss the clinical relevance of prescriptions, which is based on prospective randomized trials.

### Limitations

In order to study a homogeneous population, only managed for CCS, we imposed exclusion criteria likely to rule out profiles at high risk of bleeding (no co-prescription of anticoagulants often administered for atrial fibrillation in a more fragile and elderly population), or ischemic (ACS in the year surrounding inclusion or new angioplasty in the year following the procedure). These criteria allowed us to avoid many biases at the cost of selecting a population free of post-angioplasty ischemic complications. Thus, the ischemic risk is reduced in this selected population (exclusion of any ACS). This constitutes a bias in the selection of patients, as well as the exclusion of patients who died in the year following the procedure, but this is necessary, as our main aim was to analyse the effective duration of post-angioplasty DAPT, data collected one year after the procedure, this data is missing for patients who died. The risk of bleeding also needs to be reconsidered because of the exclusion of patients on anticoagulants. Missing or incomplete data at 1-year follow-up is a significant limitation of the study*.* The rate of prolonged prescription of DAPT is not transposable to the general population.

## Conclusions

The duration of DAPT after angioplasty for CCS in France is longer than that recommended by international guidelines, with in particular a duration of DAPT > 12 months for more than half of the patients undergoing post-angioplasty for CCS. Approximately one fifth of patients are treated with ticagrelor or prasugrel instead of clopidogrel. These results and the data in the literature on the ischemic/hemorrhagic risk related to the duration of DAPT should encourage the alignment of practices with the guidelines.

## Data Availability

The original contributions presented in the study are included in the article/**Supplementary Material**, further inquiries can be directed to the corresponding author.
