# Supplementary material for: Is the duration of dual antiplatelet therapy (DAPT) excessive in post-angioplasty in chronic coronary syndrome? Data from the France-PCI registry (2014–2019)
Source: Front Cardiovasc Med. 2023 Mar 24;10:1106503. doi: 10.3389/fcvm.2023.1106503 (PMC10080068; doi:10.3389/fcvm.2023.1106503)
Supplement: Supplementary file 1 [file Table1.docx]

**Supplementary material**

**Is the duration of dual antiplatelet therapy (DAPT) excessive in post-angioplasty in chronic coronary syndrome ? Data from the France-PCI registry (2014-2019)**

Mezier A^1*^, Motreff P^1^, Clerc JM^2^, Bar O^3^, Deballon R^4^, Demicheli T^5^,Dechery T^6^, Souteyrand G^1^, Py A^7^, Lhoest N^8^, Lhermusier T^9^, Honton B^10^, Gommeaux A^11^, Jeanneteau J ^12^, Deharo P^13^, Benamer H^14^, Cayla G^15^, Koning R^16^, Pereira B^17^, Collet JP^18^, Rangé G^5^

*** Correspondence:**

MEZIER A., MD

CHU Clermont Ferrand, 63000 Clermont Ferrand, 58 rue Montalemberg.

Phone +33614877181

Email : [amezier@chu-clermontferrand.fr](mailto:amezier@chu-clermontferrand.fr)

**Supplementary table :**

**Sensitivity analysis comparing population analysed with patients excluded from this analysis due to missing or incomplete data at 1 year follow up (4478) or death at 1 year follow up (801).**

*n= number of patients (%). SD : standard deviation ; Severe left ventricular dysfunction = LVEF : left ventricular ejection fraction <30% ; Severe renal failure* *= creatinine>200𝜇mol/L*

|  | **Population analysed**  **(8836)** | **Missing or incomplete data at 1 year follow up**  **(4478)** | **Deaths at 1 year follow up**  **(801)** |
| --- | --- | --- | --- |
| **Cardiovascular risk factors** |  |  |  |
| Age, mean (SD), year | 68.6 (+/-10.4) | 71.1 (+/- 11) | 76.5 (+/- 10.2) |
| Male gender (%) | 6857 (77.6) | 3467 (77.4) | 609 (76.0) |
| Overweight (BMI >25) (%) | 6059 (68.6) | 3028 (67,6) | 475 (59.3) |
| Hypertension (%) | 5546 (62.8) | 2896 (64.7) | 572 (71.4) |
| Dyslipidaemia (%) | 5167 (58.5) | 2340 (52.3) | 393 (49.1) |
| Smoking (%)  Active smoker (%) | 4218 (47.7)  567 (13.7) | 1971 (44.0)  687 (15.3) | 351 (43.8)  106 (13.2) |
| Diabetes (%)  Non-insulin requiring (%)  Insulin-requiring (%) | 2812 (31.9)  997 (24.0)  210 (5.1) | 1449 (32.4)  1121(25.0)  328 (7.3) | 307 (38.3)  205 (25.6)  102 (12.7) |
| Coronary heredity (%) | 2236 (25.3) | 914 (20.4) | 96 (12.4) |
| **Cardiovascular history** |  |  |  |
| Coronary angioplasty (%) | 3440 (38.9) | 1149 (25.7) | 321 (40.1) |
| Myocardial infarction (>1 year) (%) | 1322 (15.0) | 583 (13.0) | 154 (19.2) |
| Stroke (%)  Hemorrhage **(%)** | 262 (3.0)  10 (0.2) | 214 (4.8)  8 (0.2) | 64 (8.0)  2 (0.2) |
| Peripheral vascular pathology (%) | 1214 (13.7) | 605 (13.5) | 172 (21.5) |
| **Severe renal failure** (%) | 153 (1.7) | 124 (2.8) | 68 (8.5) |
| **Severe left ventricular dysfunction** (%) | 287 (3.4) | 304 (6.8) | 83 (10.4) |
| **DAPT score, median** (Quartile) | 0 (0 ; 1) | 0 (-1 ; 1) | 0 (-1 ; 1) |
| **DAPT score ≥2** (%) | 1954 (22.1) | 511 (11.4) | 141 (17.6) |

**DAPT durations according to population characteristics, procedural data, in-hospital events and 1-year follow-up.** *(n= number of patients (%). SD : standard deviation ; BARC : Bleeding Academic Research Consortium ; CABG : coronary artery bypass graft ; CTO : chronic total occlusion ; Severe left ventricular dysfunction = LVEF : left ventricular ejection fraction <30% ; Severe renal failure* *= creatinine>200𝜇mol/L)*

|  | **Clopidogrel**  **n = 7159** | **Ticagrelor/Prasugrel**  **n = 1550** | **Total**  **n = 8709** | **p-value** |
| --- | --- | --- | --- | --- |
| **Cardiovascular risk factors** |  |  |  |  |
| Age, mean (SD), year | 69.4 (+/-10.3) | 64,6 (+/-10.2) | 68.7 (+/-10.4) | <0.001 |
| Male gender (%) | 5487 (76.6) | 1261 (81.4) | 6748 (77.5) | <0.001 |
| Overweight (BMI >25) (%) | 4860 (67.9) | 1111 (71.7) | 5971 (68.6) | 0.012 |
| Hypertension (%) | 4572 (63.9) | 887 (57.2) | 5459 (62.7) | <0.001 |
| Dyslipidaemia (%) | 4259 (59.5) | 830 (53.5) | 5089 (58.4) | <0.001 |
| Smoking (%)  Active smoker (%) | 3344 (46.7)  1040 (14.5) | 808 (52.1)  281 (18.1) | 4152 (47.7)  1321 (15.2) | <0.001  <0.001 |
| Diabetes (%)  Insulin-requiring (%) | 2264 (31.6)  480 (6.7) | 507 (32.7)  101 (6.5) | 2771 (31.8)  581 (6.7) | 0.41  0.78 |
| Coronary heredity (%) | 1805 (25.2) | 397 (25.6) | 2202 (25.3) | 0.41 |
| **Cardiovascular history** |  |  |  |  |
| Coronary angioplasty (%) | 2694 (37.6) | 694 (44.8) | 3388 (38.9) | <0.001 |
| Myocardial infarction (>1 year) (%) | 985 (13.8) | 310 (20.0) | 1295 (14.9) | <0.001 |
| Stroke (%)  Hemorrhage (%) | 332 (4.6)  20 (0.3) | 44 (2.8)  1 (<0.1) | 376 (4.3)  21 (0.2) | <0.01  <0.001 |
| Peripheral vascular pathology (%) | 1035 (14.4) | 160 (10.3) | 1195 (13.7) | <0.001 |
| **Severe renal failure** (%) | 136 (1.9) | 16 (1.0) | 152 (1.7) | 0.018 |
| **Severe left ventricular dysfunction** (%) | 221 (3.1) | 61 (4.0) | 282 (3.2) | 0.05 |
| **DAPT score, median** (Quartile) | 0 (-1 ; 1) | 1 (0 ; 1) | 0 (0 ; 1) | <0.001 |
| **DAPT score ≥2** (%) | 1443 (20.2) | 489 (31.5) | 1932 (22.2) | <0.001 |
| **Lesion characteristics** |  |  |  |  |
| Number of vessel(s) affected (%)  Monotruncular (%)  Pluritruncal (%)  Isolated left main (%) | 2900 (40.5)  4217 (58.9)  42 (0.6) | 606 (39.1)  932 (60.1)  12 (0.8) | 3506 (40.3)  5149 (59.1)  54 (0.6) | 0.43 |
| Presence of proximal lesion (%) | 4261 (59.5) | 955 (61.6) | 5216 (59.9) | 0.13 |
| Presence of CTO (%) | 1656 (23.1) | 357 (23.0) | 2013 (23.1) | 0.93 |
| Artery diameter at lesion <3mm (%) | 4629 (64.6) | 1038 (67.0) | 5667 (65.1) | 0.008 |
| Lesion length >20mm (%) | 3141 (43.8) | 734 (47.3) | 3875 (44.5) | 0.012 |
| **Characteristics of angioplasty** |  |  |  |  |
| Number of dilated arteries >1 (%) | 1237 (17.3) | 286 (18.4) | 1523 (17.5) | 0.27 |
| Number of dilated sites >1 (%) | 2762 (38.6) | 647 (41.7) | 3409 (39.1) | 0.02 |
| Number of stents implanted >1 (%) | 2931 (40.9) | 698 (45.0) | 3629 (41.7) | <0.01 |
| Stent diameter <3mm (%) | 3492 (48.8) | 791 (51.0) | 4283 (49.2) | 0.11 |
| **In-hospital events** |  |  |  |  |
| Severe bleeding (BARC≥ 3) (%) | 13 (0.2) | 5 (0.3) | 18 (0.2) | 0.35 |
| Stroke (%) | 3 (<0.1) | 0 | 3 (<0.1) |  |
| **Events at 1 year** |  |  |  |  |
| Severe bleeding (BARC≥ 3) (%) | 99 (1.4) | 19 (1.2) | 118 (1.3) | 0.63 |
| Stroke (%)  Hemorrhage (%) | 27 (0.4)  3 (<0.1) | 3 (0.2)  0 | 30 (0.3)  3 (<0.1) | 0.26 |
